# Mechanistic Study of MiR-30c-5p Regulation of SIRT Expression in Polycystic Ovary Syndrome

**DOI:** 10.1007/s43032-025-01932-5

**Published:** 2025-08-25

**Authors:** Lifei Zhou, Bo Zheng, Yan Luo, Pingping Zhang, Fangfang Dai, Mingming Zhang, Shusong Wang, Yali Li

**Affiliations:** 1 Department of Reproductive and Genetics, Hebei General Hospital, Shijiazhuang, 050000 Hebei China; 2 Xingtai Infertility Specialist Hospital, Xingtai, 054000 Hebei People’s Republic of China; 3 Hebei Key Laboratory of Metabolic Diseases, Hebei General Hospital, Shijiazhuang, 050000 Hebei China; 4 Hebei Key Laboratory of Reproductive Medicine, Hebei Reproductive Health Hospital, Shijiazhuang, 050071 Hebei China

**Keywords:** MiR-30c-5p, SIRT1, PCOS, Ovarian granulosa cells

## Abstract

Abnormal development of granulosa cells is widely recognized as a critical factor contributing to polycystic ovary syndrome (PCOS). However, the precise etiology and underlying mechanisms of this disorder remain largely elusive. Accumulating evidence suggests that dysregulation of microRNAs (miRNAs) plays a pivotal role in the pathogenesis of PCOS. In this study, we systematically investigated the functional impact of miR-30c-5p on the human cumulus cells (CCs). Our findings revealed that miR-30c-5p suppresses the proliferation and induces apoptosis in the human granulosa-like tumor cell line (KGN) via targeting SIRT1. Notably, the expression level of miR-30c-5p was significantly elevated in PCOS patients compared to healthy controls, whereas the expression of SIRT1 was markedly reduced. A negative correlation was observed between miR-30c-5p and SIRT1 expression. Mechanistically, upregulation of miR-30c-5p led to decreased expression of SIRT1 and Bcl-2 proteins, while simultaneously enhancing the expression of Bax proteins. Furthermore, our data confirmed that SIRT1 serves as a direct target of miR-30c-5p. Collectively, these results indicate that miR-30c-5p promotes apoptosis of GCs by directly targeting SIRT1, thereby representing a novel molecular target for improving GC dysfunction in PCOS patients.

## Introduction

Polycystic ovary syndrome (PCOS) is the most prevalent endocrine and metabolic disorder, affecting 5–10% of women of reproductive age, this syndrome is characterized by high androgen levels, ovulation disorders, polycystic ovaries, and insulin resistance (IR) [1]. In addition to clinical manifestations such as irregular menstruation, infertility, obesity, and hirsutism, women with PCOS have a higher susceptibility to metabolic diseases such as cardiovascular disease, hypertension,and diabetes [2–4]. Although lifestyle modifications,hormone therapy, and technologies such as in vitro fertilization and embryo transfer (IVF-ET) have notably improved pregnancy rates, outcomes still do not meet expectations. PCOS is influenced by genetic, environmental, and hormonal factors that significantly contribute to its development. Recent studies have highlighted the critical role of microRNAs (miRNAs) in the pathogenesis of PCOS, although the sequencing results have yet to be further validated in clinical samples and the exact mechanism is unclear [5].

In the study of PCOS, granulosa cells (GCs) surrounding oocytes play a role not only in regulating follicular growth through the secretion of hormones and cytokines but also in other aspects [6]. These cells not only provide nutrients and oxygen to support the normal maturation of developing oocytes but also produce a series of signaling molecules that promote or inhibit follicular growth while interacting with other cells involved in follicle development [7–9]. However, abnormal function of GCs can cause various issues. For example, GC dysfunction in patients with PCOS may lead to impaired follicular development, ovulatory disorders, and infertility [10]. This is because the dysfunctional GCs do not provide adequate support and regulation to the developing oocyte. In addition, recent studies have confirmed these findings, observing that GC dysfunction in PCOS is associated with decreased cellular proliferation and increased apoptosis, which contribute to follicular growth atresia [11].

MiRNAs are a class of short-stranded non-coding RNAs that regulate the expression of transcribed genes during tissue differentiation and development, thereby impacting cellular function and metabolic status. Research has indicated that the abnormal expression of miRNAs is linked to the pathological progression of various ailments, including tumors, reproductive disorders, and metabolic disorders [12]. miR-30c-5p acts as a tumor suppressor in several malignancies, such as ovarian, endometrial, and papillary thyroid cancers, influencing cancer cell proliferation, migration, apoptosis, and angiogenesis [13–15]. Furthermore, some researchers have found that miR-30c-5p levels are significantly elevated in the blood, follicular fluid, and GCs of patients with PCOS, identified through sequencing [16–18]. However, this sequencing result has not been further validated using clinical samples, and the underlying mechanisms remain to be elucidated.

Sirtuin 1 (SIRT1), a deacetylase predominantly located in the nucleus, alters the function and localization of cellular proteins. Initially studied extensively as a longevity gene, its association with fertility defects was first discovered in 2003. SIRT1 regulates mitochondrial biogenesis, defense against oxidative stress, and energy homeostasis, thereby enhancing GCs proliferation and spermatogenesis [19, 20]. Some researchers have found that overexpression of miR-23a inhibited the expression of SIRT1 and promoted apoptosis in human GCs, which not only affected the function of SIRT1, but also further triggered a series of intracellular signaling pathway changes, and this discovery revealed the important role of SIRT1 in the regulation of cellular [21]. Bioinformatics analysis has revealed complementary pairing between miR-30c-5p and the 3ʹ-untranslated region (UTR) of SIRT1 mRNA. However, the role of miR-30c-5p in regulating SIRT1 and its impact on the development of PCOS remain unclear. Therefore, this study aimed to explore the differential expression of miR-30c-5p and SIRT1 in GCs obtained from individuals with PCOS, as well as their role and significance in apoptosis. The findings from this research contribute to our understanding of the underlying mechanisms of abnormal proliferation and apoptosis in GCs associated with PCOS, offering valuable insights for both diagnosis and treatment approaches for PCOS.

## Materials and Methods

### Patients and GC Extraction

This study included 44 participants (23 patients with polycystic ovary syndrome and 21 control patients) who underwent intracytoplasmic sperm injection (ICSI) or in vitro fertilization (IVF) at the Xingtai Fertility Hospital's Center for Reproductive Medicine, employing the GnRH agonist long protocol or antagonist regimen. The Institutional Review Board of the Center for Reproductive Medicine, Xingtai Fertility Hospital, approved this study(202,401). The controls consisted of 23 individuals diagnosed with PCOS based on the 2003 Rotterdam criteria [22], alongside individuals who, despite their regular menstrual cycles and normal ovarian function, experienced infertility owing to tubal or male factors. Patients aged over 35 years, as well as those with endometriosis, premature ovarian insufficiency, reproductive system tumors, immune disorders, a history of radiotherapy or chemotherapy, hypertension, diabetes, or chromosomal abnormalities were excluded from the study. GCs were isolated from the limpid follicular fluid (FF) collected on the day of oocyte retrieval which were immediately stored at −80 °C for subsequent quantitative real-time polymerase chain reaction (qRT-PCR) and western blot analysis.

### qRT-PCR Analysis

The miRNA and mRNA from the patients were extracted using kits from TianGen (China), and the RNA concentration and purity were assessed and found satisfactory. Subsequently, the RNA was converted to cDNA using the corresponding kits (TianGen, China), respectively. qRT-PCR (Life Technologies, Singapore) was performed under the following reaction conditions: an initial pre-denaturation at 95 °C for 15 min, followed by 40 cycles of denaturation at 95 °C for 10 s, annealing at 60 °C for 20 s, and extension at 72 °C for 32 s. The relative expression levels of miRNA and mRNA were quantified using the 2^−ΔΔCT^ method, with GAPDH serving as the internal reference. Primers for miR-30c-5p and SIRT1 were designed and synthesized by RUIBO, while the forward primer for GAPDH was 5’-GGAGCGAGATCCCTCCAAAAT-3’, and the reverse primer was 5’-GGCTGTTGTCATACTTCTCATGGG-3’, both synthesized by TongYong (China).

### Dual Luciferase Assay

SIRT1 was predicted to be a target of miR-30c-5p by TargetScan (https://www.targetscan.org/vert) and miRDB (https://mirdb.org/). Wild-type (WT) and mutant (MUT) sequences of the SIRT1 3′-UTR target site were synthesized and then cloned into the pmirGLO vector, and this reaction system was placed at 22 °C for 1 h. The KGN cells were seeded into 96-well plates and cultured overnight at 37 °C in an atmosphere containing 5% CO_2_ until transfection for 48 h. The following experimental groups were established: a blank group, pmirGLO + NC, pmirGLO + hsa-miR-30c-5p, SIRT1-WT + NC, SIRT1-WT + hsa-miR-30c-5p, SIRT1-MUT + NC, and SIRT1-MUT + hsa-miR-30c-5p. Each group consisted of three replicate wells, with 200 ng of plasmid per well and a final miRNA concentration of 100 nM per well. After 36 h of transfection, luciferase activity was measured using the Dual-Luciferase® Reporter Assay System (Promega, Madison, WI, USA).

### Western Blotting

The cells were treated with RIPA lysate containing protease inhibitors (Solarbio, Beijing, China) to extract total protein. After centrifugation, the resulting supernatant was utilized for the separation of proteins via sodium dodecyl sulfate–polyacrylamide gel electrophoresis (SDS-PAGE), followed by their transfer onto polyvinylidene difluoride (PVDF) membranes. The membranes were blocked in 5% skim milk powder for 120 min and then incubated overnight at 4 °C with primary antibodies SIRT1 (San Ying, 1:1500 dilution, Wuhan, China), GAPDH (San Ying, 1:15,000 dilution, Wuhan, China), Bax (GB15690, Servicebio, China, 1:1000 dilution), Bcl-2 (GB154380, Servicebio, China, 1:1000 dilution), and β-actin (GB15003, Servicebio, China, 1:5000 dilution). This was followed by incubation with a horseradish peroxidase-labeled sheep anti-rabbit secondary antibody (ZhengNeng, 1:10,000 dilution, China) at room temperature for 1 h. Protein bands were visualized using an enhanced chemiluminescence detection reagent and quantified densitometrically with ImageJ software(v.1.46, National Institute of Health of Bethesda, Bethesda, MD, USA).

### Cell Culture and Transfection

The human ovarian granulosa cell line (KGN) was generously provided by the Hebei Maternal and Child Health Institute, China. These cell lines were cultured in DMEM/F12 (Gibco, USA) supplemented with 9% fetal bovine serum (Viva Cell, Shanghai, China) and 1% penicillin/streptomycin (Solarbio, Beijing, China). Cultivation was performed at 37 °C in an incubator with a 5% CO_2_ atmosphere.

KGN cells were divided into four groups: miR-30c-5p mimics, miR-30c-5 mimic NC miR-30c-5 inhibitor, and miR-30c-5p inhibitor NC (RUIBO, China). The miR-30c-5p mimics, miR-30c-5 mimics NC, miR-30c-5 inhibitor, and miR-30c-5p inhibitor NC were transfected using Lipofectamine 2000 reagent (Invitrogen, Carlsbad, CA, USA). After replacing the complete medium with DMEM (Gibco, USA), the cells were incubated at 37 °C in a 5% CO_2_ atmosphere for 6 h, after which the complete medium was replaced.

### Cell Counting Kit-8 (CCK-8) Assay

Transfected granulocytes were seeded into 96-well plates at a density of 1500 cells/well. Four replicate plates were prepared to evaluate cell proliferation at 0 h, 24 h, 48 h, and 72 h, respectively. Each group within the plates consisted of five parallel wells. To each well, 100 µL of DMEM/F12 basal medium was added before placing the plates in a cell culture incubator for continued cultivation. After incubation, 10 µL of CCK-8 reagent (Shanghai, China) was added to each well, and the OD at 450 nm was measured using an enzyme marker.

### Cell Apoptosis Assay

Forty-eight hours post-transfection, KGN cells were harvested using trypsin (Solarbio, Beijing, China), washed twice with 2 mL of pre-cooled sterile phosphate-buffered saline (PBS), and resuspended in the same buffer to a concentration of 1 × 10^6^ cells/mL. The 5 × Binding Buffer (MULTI SCIENCES, China) was diluted to a 1 × working solution using double-distilled water, and 500 µL of this solution was used to resuspend the cells. Next, 5 µL of Annexin V-FITC and 10 µL of propidium iodide (PI) (both from MULTI SCIENCES, China) were added to the cell suspension. The mixture was incubated for 5 min in the dark at room temperature. Apoptotic cells were then detected using a flow cytometer with FITC and PI detection channels at excitation/emission wavelengths of 488 nm/530 nm and 535 nm/615 nm, respectively. The apoptosis rate was calculated as the sum of early and late apoptosis rates.

### Statistical Analysis

Statistical analysis was performed using SPSS software (version 22), with data expressed as mean ± standard deviation (SD). The Kolmogorov–Smirnov test was used to assess the normality of data distribution. Statistical significance was determined using the Student's t-test for normally distributed variables and the Mann–Whitney U test for non-parametric tests. *P* < 0.05 was considered an indicator of statistical significance.

## Results

### Clinical and Endocrine Indices in Patients with PCOS and Controls

The differences in age, estrogen (E2), and progesterone (P) between the two groups were not statistically significant (*P* > 0.05). Body mass index (BMI), basal luteinizing hormone (LH), and testosterone (T) levels of the PCOS group were significantly higher than those of the control group, however, follicle stimulating hormone and prolactin levels of the PCOS group were significantly lower than those of the control group, and the differences were statistically significant (*P* < 0.05).

### miR-30c-5p Expression is Elevated in GCs of Patients with PCOS

**Fig. 1:**
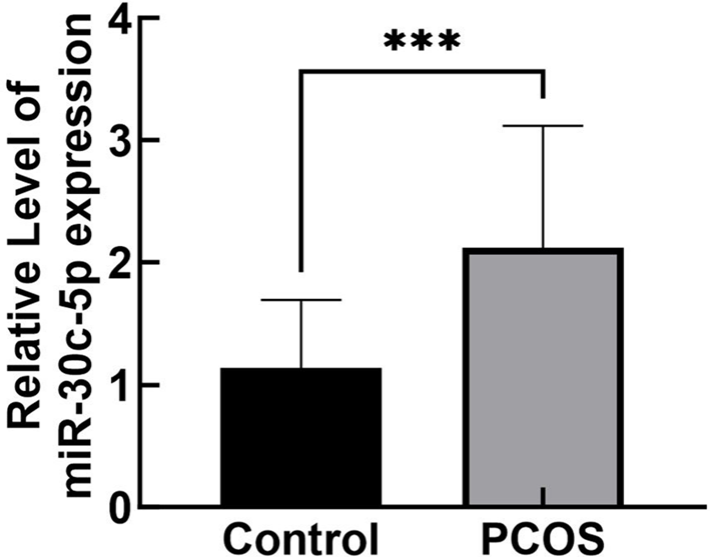
Expression of miR-30c-5p in GCs of PCOS patients. miR-30c-5p expression was elevated in GCs of PCOS patients,***P < 0.001

The miRNA was extracted from the GCs of patients in both groups, and the expression level of miR-30c-5p was quantified by qRT-PCR. The results showed that the expression of miR-30c-5p was significantly increased in the PCOS group compared with the control group (*P* < 0.05) (Fig. 1A).

### SIRT1 is a Predicted Target of miR-30c-5p

**Fig. 2:**
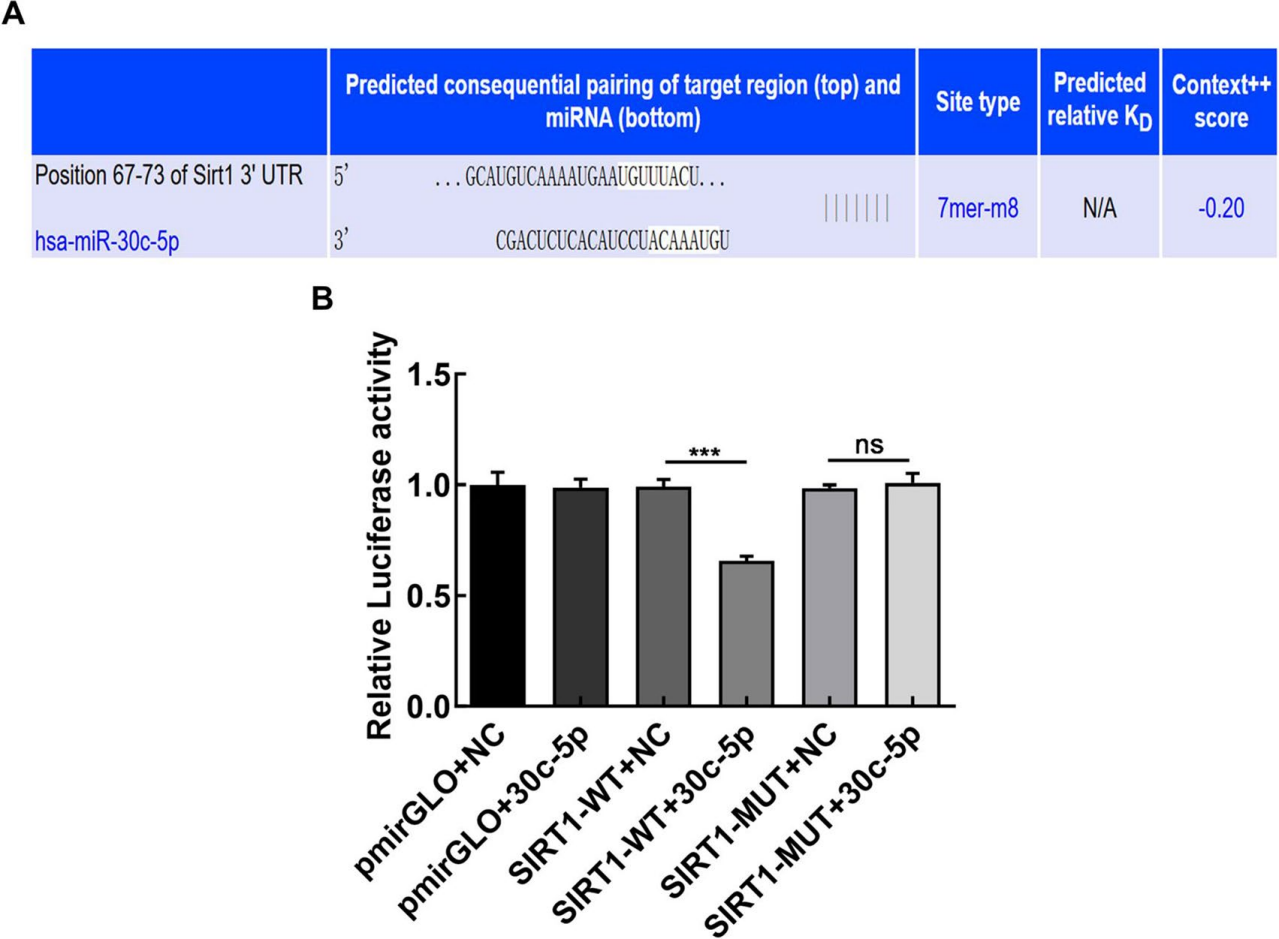
**A** SIRT1 is a regulatory target of miR-30c-5p **B** A dual luciferase reporter assay was used to confirm the relationship between miR-30c-5p and SIRT1. Data are expressed as mean ± SD; Statistical analysis was carried out using Student's t-tests. ***P < 0.001; *NS*, not significant

**Fig. 3:**
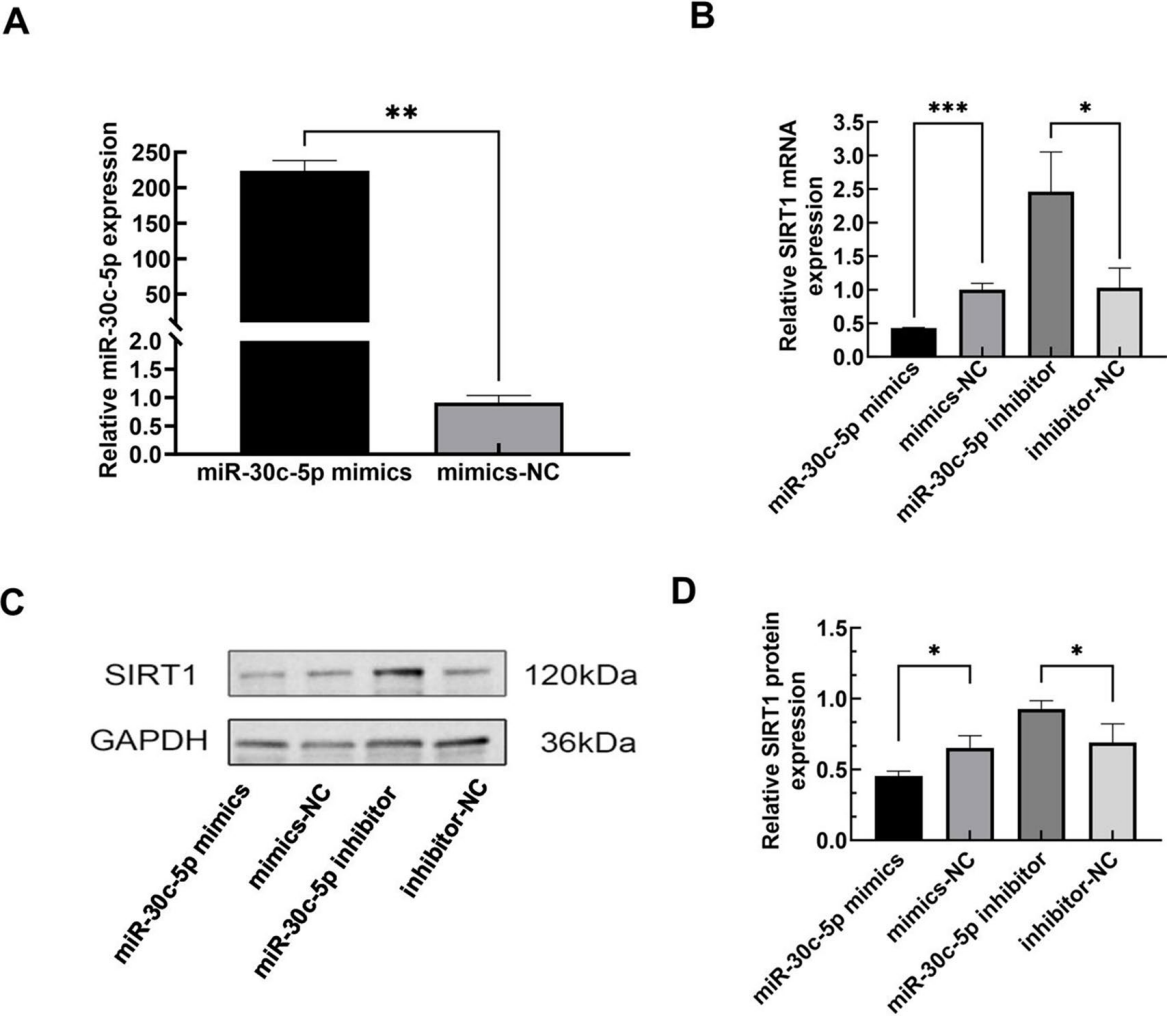
SIRT1 is a direct target of miR-30c-5p. **A** Levels of miR-30c-5p in each group after transfection, as detected by qRT-PCR. **B** SIRT1 mRNA expression was assessed in cells transfected with miR-30c-5p overexpression or knockdown using qRT-PCR analysis. **C**-**D** SIRT1 protein expression was assessed in cells transfected with miR-30c-5p overexpression or knockdown using protein blotting analysis. *P < 0.05 compared with the corresponding negative control

To elucidate the potential mechanism of miR-30c-5p in PCOS, we initially utilized TargetScan, miRDB, mirtarbase, and miRWalk websites to predict the target genes of miR-30c-5p. Both TargetScan and miRDB websites predicted that miR-30c-5P exhibits a complementary pairing sequence with the SIRT1 3ʹ-UTR region with complementary paired sequences (2A). SIRT1-WT + hsa-miR-30c-5p exhibited a significant decrease in luciferase activity compared with the SIRT1-WT + NC group, suggesting that hsa-miR-30c-5p may target SIRT1. Conversely, SIRT1-MUT + hsa-miR-30c-5p did not display significant changes in luciferase activity compared with the SIRT1-MUT + NC group, suggesting that the mutation site is critical for miR-30c-5p targeting SIRT1 (Fig. 2B). Furthermore, to investigate the regulatory role of miR-30c-5p on SIRT1 expression, we performed both qRT-PCR and western blotting. We established KGN cell lines stably expressing high levels of miR-30c-5p through transfection with a miR-30c-5p mimics, as confirmed in the graph (Fig. 3A). Our results suggest that miR-30c-5p mimics downregulated the mRNA and protein levels of SIRT1, whereas inhibitors of miR-30c-5p upregulated the mRNA and protein levels of SIRT1 (Fig. 3B, C, D). These results suggest that miR-30c-5p may exert its effects through SIRT1 expression.

### SIRT1 Expression is Decreased in GCs of Patients with PCOS

**Fig. 4:**
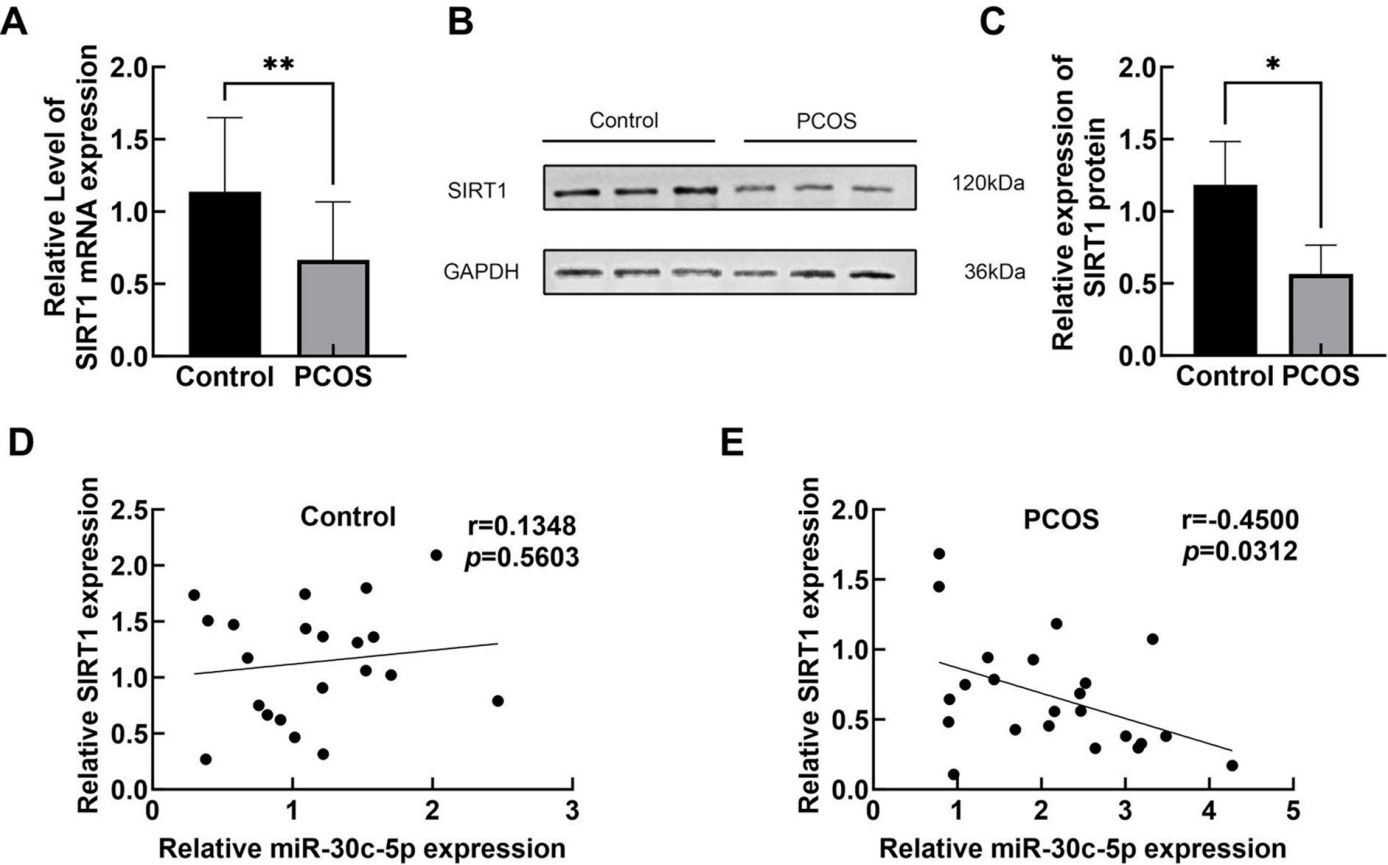
Expression of SIRT1 in GCs of PCOS patients. **A** SIRT1 mRNA expression was decreased in GCs of PCOS patients. **B**-**C** Protein expression of SIRT1 was decreased in PCOS patients. **D**-**E** Evaluated by using Spearman's correlation analysis. The correlation between miR-30c-5p and SIRT1, *P < 0.05 compared with the non-PCOS group

Tthe expression level of SIRT1 mRNA was reduced in the GCs of patients with PCOS compared with controls (Fig. 4A). These results were consistent with those observed at the protein level (Fig. 4B, C). A Spearman correlation analysis was conducted to explore the relationship between miR-30c-5p and SIRT1 mRNA. The results showed that SIRT1 mRNA was negatively correlated with the expression of miR-30c-5p in the GCs of patients with PCOS(*r* = −0.4500, *P* = 0.0312), however, this correlation was not found in the control group (*r* = 0.1348, *P* = 0.5603) (Fig. 4D, E).

### miR-30c-5p Inhibits Cell Proliferation and Promotes Apoptosis

**Fig. 5:**
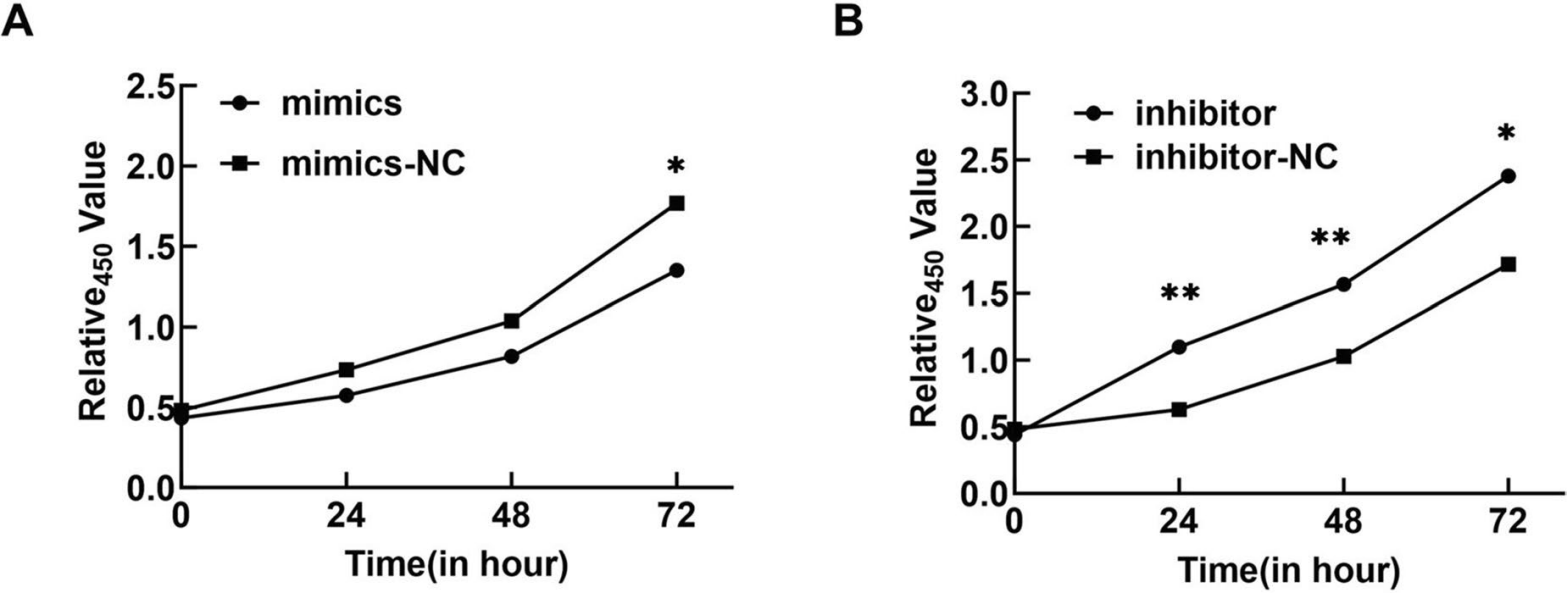
miR-30c-5p inhibits cell proliferation and promotes apoptosis. **A** levels of miR-30c-5p in each group after transfection, detected by qRT-PCR. **B** cell proliferation was performed using CCK-8 assay at 24, 48, and 72 h after cell transfection, *P < 0.05

**Fig. 6:**
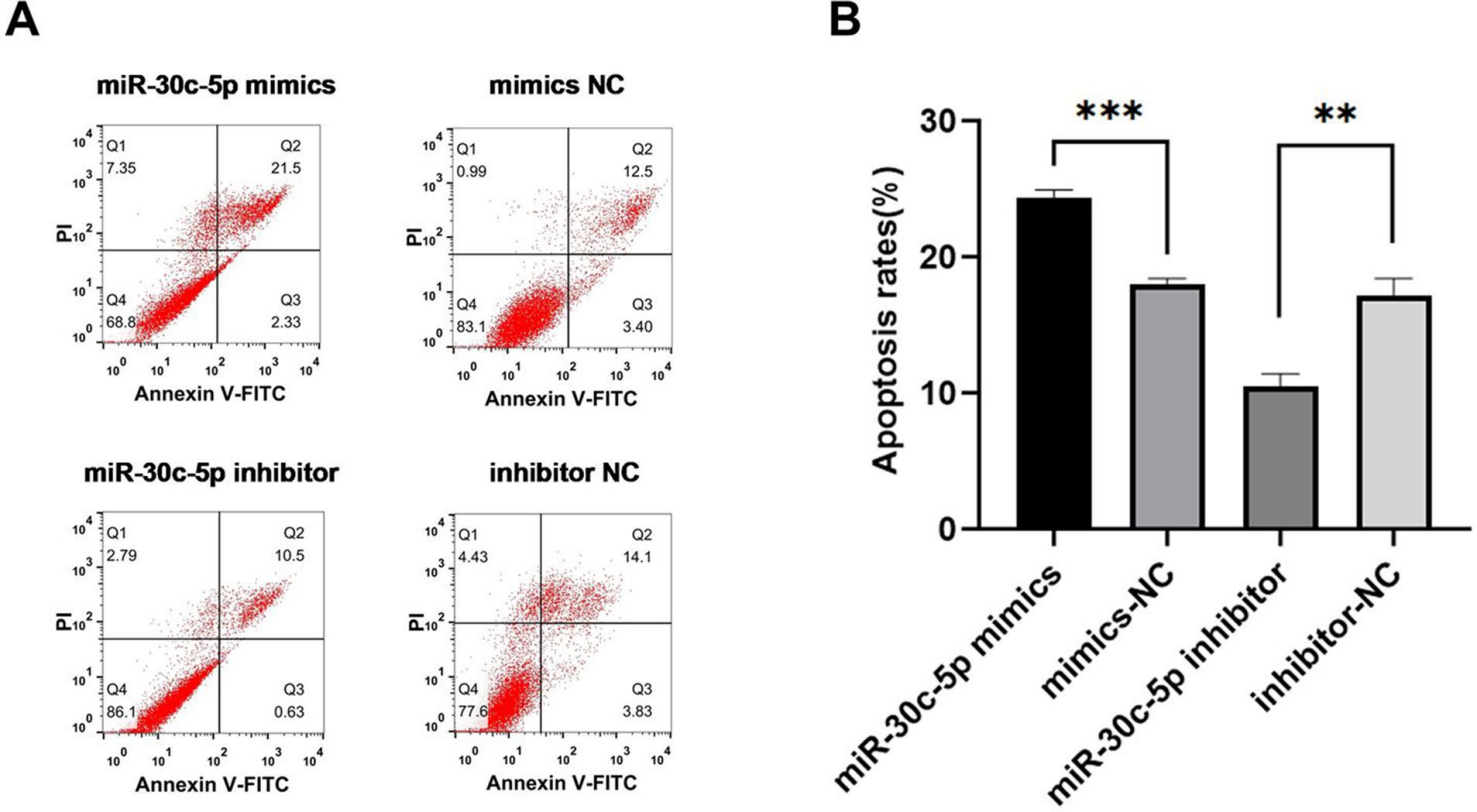
**A**-**B** apoptosis rates of KGN cells in each group were detected by flow cytometry, miR-30c-5p mimics: miR-30c-5p overexpression; miR-30c-5p inhibitor: miR-30c-5p suppression; **P < 0.01, ***P < 0.001

**Fig. 7:**
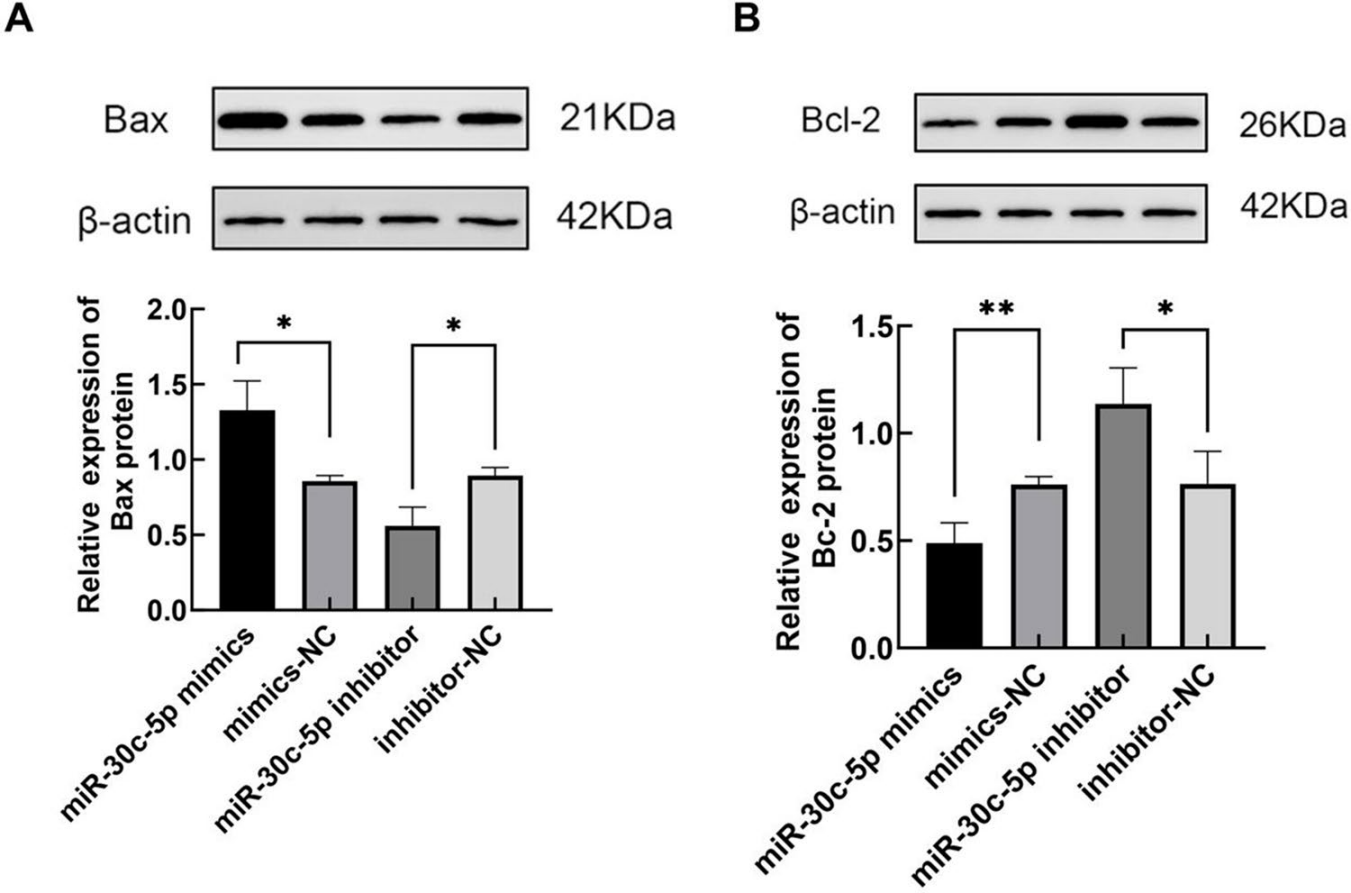
**A**-**B** Bax and Bcl-2 protein expression. Compared with the corresponding negative control, *P < 0.05

CCK-8 experiments showed that cell viability in the miR-30c-5p mimic group decreased at 72 h post-transfection compared with cells in the miR-30c-5p mimic NC. In contrast, cells transfected with the miR-30c-5p inhibitor exhibited higher viability than those in the miR-30c-5p inhibitor NC group at 24 h, 48 h, and 72 h after transfection (Fig. 5A, B). Flow cytometry assays showed that miR-30c-5p increased the apoptosis rate in GCs compared with the miR-30c-5p mimic NC group, whereas the miR-30c-5p inhibitor group showed the opposite effect (Fig. 6A, B). Furthermore, the miR-30c-5p mimic group exhibited upregulation of Bax expression and downregulation of Bcl-2 expression. The reverse pattern was observed in the miR-30c-5p inhibitor group (Fig. 7A, B). The results indicate that miR-30c-5p inhibits the proliferation of KGN cells and promotes apoptosis.

## Discussion

The objective of this research was to explore the potential role of miR-30c-5p in the atypical proliferation of GCs in PCOS and to elucidate its underlying mechanisms. Our results showed that miR-30c-5p expression was significantly elevated in the GCs of infertile women with PCOS, while SIRT1 expression was significantly decreased and negatively correlated with miR-30c-5p levels in these patients. These findings validate our initial hypothesis. To verify the relationship between miR-30c-5p and SIRT1, we transfected KGN cells with miR-30c-5p mimics, miR-30c-5p inhibitors, and their respective NCs. The results showed that transfection with miR-30c-5p mimics resulted in decreased mRNA and protein expression of SIRT1 in KGN cells, whereas the miR-30c-5p inhibitor elevated both mRNA and protein levels of SIRT1 in KGN cells. Furthermore, miR-30c-5p directly targeted the 3ʹ-UTR of SIRT1 mRNA. Additionally, we observed decreased cell proliferation and increased apoptosis, including elevated Bax expression and decreased Bcl-2 expression, in the miR-30c-5p mimics group. On the contrary, the miR-30c-5p inhibitor group showed increased cell proliferation, decreased apoptosis rate, decreased Bax expression, and elevated Bcl-2 expression. Our findings provide evidence that miR-30c-5p can regulate the proliferation of KGN cells and promote apoptosis through the SIRT1 pathway in GCs.

In recent years, an increasing number of studies have shown that the dysregulated role of miRNAs is a significant etiological factor in the pathogenesis of PCOS [23]. MiR-96-5p is downregulated in the serum and GCs of patients with PCOS and polycystic mice. This downregulation inhibits the synthesis of estrogen and suppresses GC proliferation by targeting FOXO1 [24]. Du team has found that miR-424 inhibits proliferation and promotes apoptosis in human ovarian GCs by directly targeting and inhibiting the expression of Apelin and APJ [25]. MiR-874-3p expression is upregulated in PCOS and promotes testosterone-induced apoptosis of GCs through inhibition of HDAC1-mediated P53 deacetylation [26]. Therefore, miRNAs can influence the occurrence and development of PCOS through various pathways. Regarding miR-30c-5p, which is located on the short arm of chromosome 1 and the long arm of chromosome 6, most studies have focused on tumors and vascular smooth muscle, with relatively few investigations in PCOS [27]. Hu team has found that miR-30c-5p inhibits oxidized LDL-induced inflammatory responses and apoptosis in human umbilical cord by downregulating the PTEN axis [28]. Another investigator found that lidocaine inhibits proliferation and cisplatin resistance in cutaneous squamous cell carcinoma by regulating the miR-30c targeting of the SIRT1 pathway [29]. In addition, upregulation of miR-30c-5p inhibits the growth, apoptosis, and migration of glioma cells by targeting Bcl-2 [30]. Zheng has reported that decreased miR-30c-5p expression promotes hepatocellular carcinoma progression by targeting RAB32 gene expression [31]. In recent years, miR-30c-5p has been found to be involved in the regulation of reproductive processes, including endometrial repair, endometriosis and sperm quality [32], by targeting multiple signaling pathways. miR-30c-5p can regulate the TGF-β/SMAD pathway to repair endometrial damage and enhance fertility in injured animals [33], and is a potential minimally invasive biomarker for endometriosis. marker with high expression in women with endometriosis diagnosed by laparoscopy [34]. In addition, some researchers suggest that miR-30c-5p may be involved in the pathogenesis of PCOS based on sequencing and bioconfidence predictions. Another study reported that the level of miR-30c-5p is increased in the ovaries of polycystic mice, aligning with our findings in patients with polycystic ovaries [35, 36]. However, the specific mechanism of its action has not yet Table 1 been clarified.

**Table 1:** Comparison of general clinical data between polycystic group and normal control group

Basic parameters	Control(*n* = 21)	PCOS(*n* = 23)	*P-*Value
Age/(years)	28.86 ± 3.38	27.91 ± 3.69	0.405
BMI/(kg/m^2^)	25.13 ± 4.05	26.85 ± 2.13	0.003*
Basal FSH(IU/L)	5.87 ± 1.02	5.79 ± 1.70	0.040*
Basal E2(pg/ml)	32.50(24.15,46.55)	35.10(26.42,46.60)	0.503
Basal P(ng/ml)	0.37(0.24,0.48)	0.48(0.35,0.58)	0.095
Basal PRL(ng/ml)	14.20(12.30,22.00)	11.00(7.71,16.10)	0.024*
Basal LH(IU/L)	3.81(3.07,5.60)	7.01(4.68,10.50)	0.007*
Basal T(ng/ml)	20.00(20.00,30.20)	34.00(25.70,46.00)	0.001*

The data are represented as mean ± SD for normally distributed variables and as median with interquartile range for non-normally distributed variables. Statistical significance was defined as *P* < 0.05, *P < 0.05

SIRT1 plays a crucial role in cell proliferation, autophagy, IR, and oxidative stress and serves as an important reproductive signaling pathway and regulator within the hypothalamic-pituitary-ovarian (HPO) axis [37]. SIRT1 inhibits GC apoptosis by activating both the extracellular signal-regulated kinase1/2(ERK1/2)and NF-kappa B(NF-κB)signaling pathways [38]. Naringin supplementation improves IR and hormone levels, attenuates pathological changes in ovarian tissues, and increases SIRT1 expression in polycystic rats [39]. Huo et al. observed that resveratrol promotes follicular development in PCOS rats by upregulating SIRT1 expression via the glycolytic pathway [40]. Contrarily, another study found that while the expression of SIRT1 is significantly increased in patients over 40 years of age, it is reduced in patients with PCOS [41]. Consistent with these studies, our present study revealed a reduction in SIRT1 expression in GC tissues of patients with PCOS. Moreover, we found that the overexpression of miR-30c-5p inhibits SIRT1 expression.

The aim of this study was to elucidate the functions of miR-30c-5p and SIRT1, as well as their relationship in the GCs of patients with PCOS. However, our study has some limitations. Firstly, the cells used were derived from cell lines, which cannot fully represent the cellular status of patients with PCOS, did not take into account the possible bias caused by not accounting for the heterogeneity of the patients and their potential confounders such as BMI, T levels, and LH/FSH ratios. Secondly, the experimental findings were not verified in animal models. Therefore, future experimental designs should include increasing the number of samples and constructing animal models to more thoroughly investigate the correlation between miR-30c-5p, SIRT1, and PCOS.

## Summary

We observed that miR-30c-5p expression was significantly elevated in patients with PCOS and negatively correlated with reduced SIRT1 levels, potentially impacting follicle development and oocyte quality in patients. In addition, cellular experiments demonstrated that overexpression of miR-30c-5p promoted apoptosis in KGN cells and inhibited the proliferation of GCs by targeting SIRT1. These findings suggest that miR-30c-5p may play a role in the pathophysiological mechanisms and progression of PCOS. These results provide a novel target for the treatment of PCOS.

## Data Availability

The data is available in reasonable circumstances.
